# Microbiota-Derived Extracellular Vesicles as a Postbiotic Strategy to Alleviate Diarrhea and Enhance Immunity in Rotavirus-Infected Neonatal Rats

**DOI:** 10.3390/ijms25021184

**Published:** 2024-01-18

**Authors:** Sergio Martínez-Ruiz, Yenifer Olivo-Martínez, Cecilia Cordero, María J. Rodríguez-Lagunas, Francisco J. Pérez-Cano, Josefa Badia, Laura Baldoma

**Affiliations:** 1 Departament de Bioquímica i Fisiologia, Facultat de Farmàcia i Ciències de l’Alimentació, Universitat de Barcelona, 08028 Barcelona, Spain; sergio_martinez_ruiz@ub.edu (S.M.-R.); yeni_olivo@hotmail.com (Y.O.-M.); ccalday@gmail.com (C.C.); mjrodriguez@ub.edu (M.J.R.-L.); franciscoperez@ub.edu (F.J.P.-C.); josefabadia@ub.edu (J.B.); 2 Institut de Biomedicina de la Universitat de Barcelona (IBUB), 08028 Barcelona, Spain; 3 Institut de Recerca Sant Joan de Déu (IRSJD), 08950 Barcelona, Spain; 4 Nutrition and Food Safety Research Institute (INSA-UB), 08921 Santa Coloma de Gramenet, Spain

**Keywords:** rotavirus, extracellular vesicles, microbiota–host communication, probiotics, postbiotics, immunomodulation, intestinal serotonin

## Abstract

Rotavirus (RV) infection is a major cause of acute gastroenteritis in children under 5 years old, resulting in elevated mortality rates in low-income countries. The efficacy of anti-RV vaccines is limited in underdeveloped countries, emphasizing the need for novel strategies to boost immunity and alleviate RV-induced diarrhea. This study explores the effectiveness of interventions involving extracellular vesicles (EVs) from probiotic and commensal *E. coli* in mitigating diarrhea and enhancing immunity in a preclinical model of RV infection in suckling rats. On days 8 and 16 of life, variables related to humoral and cellular immunity and intestinal function/architecture were assessed. Both interventions enhanced humoral (serum immunoglobulins) and cellular (splenic natural killer (NK), cytotoxic T (Tc) and positive T-cell receptor γδ (TCRγδ) cells) immunity against viral infections and downregulated the intestinal serotonin receptor-3 (HTR3). However, certain effects were strain-specific. EcoR12 EVs activated intestinal *CD68*, *TLR2* and *IL-12* expression, whereas EcN EVs improved intestinal maturation, barrier properties (goblet cell numbers/mucin 2 expression) and absorptive function (villus length). In conclusion, interventions involving probiotic/microbiota EVs may serve as a safe postbiotic strategy to improve clinical symptoms and immune responses during RV infection in the neonatal period. Furthermore, they could be used as adjuvants to enhance the immunogenicity and efficacy of anti-RV vaccines.

## 1. Introduction

Rotavirus (RV) is a non-enveloped, double-stranded RNA (dsRNA) member of the Reoviridae family that infects enterocytes in the tip of the small intestine, altering fluid secretion and absorptive function [1,2]. RV infection is a major cause of acute gastroenteritis in children under 5 years old worldwide, and is associated with high rates of mortality, principally in low-income countries, caused by excessive loss of fluids through severe diarrhea and vomiting [3,4]. Several non-inflammatory mechanisms contribute to RV-induced watery diarrhea, which include imbalances in intestinal osmosis derived from the loss of absorptive cells, effects of RV enterotoxins on chloride secretion, and activation of the enteric nervous system and neurotransmitters, such as serotonin (5-hydroxytriptamine, 5-HT) [1,5,6].

Serotonin is a crucial mediator of gut functions, having autocrine and paracrine actions acting on several receptors. In the gut, serotonin modulates vagal reflexes, gut motility and barrier permeability [7]. More than 90% of the body’s serotonin is produced by the enterochromaffin cells of the intestinal epithelium from dietary tryptophan, which is converted into 5-hydroxytryptophan by the enzyme tryptophan hydroxylase 1 (TPH1) and subsequently transformed into serotonin by a decarboxylase reaction. Free intestinal serotonin levels are controlled by the serotonin transporter (SERT), located at the apical and the basolateral sides of the cell membrane. Once released, extracellular serotonin can be taken up by intestinal epithelial cells through the SERT, and further inactivated by monoamine oxidase, the first enzyme of the serotonin degradation pathway. Serotonin exerts its effects by interacting with specific receptors of the surrounding epithelial, immune and neural cells. There are several families of 5-HT receptors (HTR), and five of them are expressed in the gut [7]. There is evidence that RV activates serotonin secretion via the enterochromaffin cells of the small intestine and that HTR3 is involved in the RV-derived activation of vomiting and diarrhea [6,8,9].

Immunity against RV infection involves innate and adaptative responses. Viral antigens are presented to B and T lymphocytes by dendritic cells and macrophages. Natural killer (NK) lymphocytes are the first line of defense against the virus, and T cytotoxic (Tc) cells also help lysis of infected cells. Finally, B cells produce antibodies (Ab) that confer long-term protection. Immunoglobulin (Ig) A seems to have a critical role in the establishment of immunity against RV infection [10,11,12]. Although the introduction of oral RV vaccines into global vaccination programs has improved the health burden of RV diarrhea in children, their implementation and efficacy in underdeveloped countries is still limited. In these countries, challenges include the high costs of vaccination programs and the reduced efficacy of the vaccines, most likely due to low standards of hygiene, suboptimal breastfeeding, malnutrition and derived gut microbiota dysbiosis [13,14,15,16]. For this reason, new strategies to enhance immunity against both RV infection and vaccines or to ameliorate RV-induced diarrhea are needed. 

In this context, interventions with probiotic strains of the *Bifidobacterium*, *Lactobacillus* and *Escherichia* genera have been explored in several neonatal animal models (mouse, rat, piglet) to prove their efficacy in improving immunity against RV and ameliorating diarrhea and clinical markers [17,18,19,20,21,22,23]. Comparative studies revealed that the probiotic *E. coli* Nissle 1917 (EcN) is more effective than Gram-positive probiotics in enhancing protective immunity against RV [24].

The probiotic EcN is a good colonizer of the human gut and positively influences gastrointestinal homeostasis and microbiota balance [25,26]. Clinical trials have proved its therapeutic benefits in the remission of inflammatory bowel diseases [27] and acute diarrhea in children [28]. In preclinical assays using gnotobiotic neonatal pigs, colonization with EcN has been shown to efficiently protect against RV infection through several mechanisms that involve the protection of the intestinal epithelial barrier, stimulation of the innate immune system and interference with pathogen binding to intestinal epithelial cells [18,21,22]. Recent studies also prove the beneficial effects of EcN against RV-induced diarrhea and immune responses in a neonatal malnourished pig model colonized with human infant fecal microbiota [29,30]. In addition, colonization of gnotobiotic pigs with probiotic EcN enhances the immunogenicity and protective efficacy of an oral attenuated RV vaccine [31]. 

Although probiotics are generally recognized as safe (GRAS) biological agents or Qualified Presumption of Safety (QPS) according to the European Food Safety Authority, some concerns about the potential risk of bacterial translocation should be considered, especially in critically immunocompromised individuals and neonates [32,33]. In this context, postbiotics are now being considered as a new health-promoting strategy. Postbiotics are probiotic- or microbiota-derived components that confer a health benefit on the host, including bacterial cell fragments, biomolecules and secreted bioactive compounds [34]. This approach overcomes the disadvantages associated with the administration of live bacteria. Recent studies show that postbiotics could improve intestinal homeostasis and prevent enteric infections [35,36]. 

Considering the definition provided by the International Scientific Association for Probiotics and Prebiotics [34], bacterial extracellular vesicles (EVs) fit the postbiotic definition as they are cell-inanimate structures, produced from the bacterial cell membrane, that cannot replicate and mediate beneficial effects on the host. In general, bacterial EVs are spherical membrane-derived nanostructures (typically ranging from 25 to 300 nm in diameter) that contain proteins, lipids, nucleic acids, numerous surface and cytosolic biomolecules, and metabolites produced from their progenitor bacteria. The vesicle components depend on the producer strain, its physiological state and environmental conditions [37,38]. Bacterial EVs, as carriers of biological information, are important messengers in interspecies communication, either between members of the same microbial community or between bacteria and the host [39]. In the gut, microbiota-released EVs diffuse through the mucin layer, interact with epithelial and immune cells of the intestinal mucosa and deliver the cargo molecules upon internalization into target cells. The delivered bacterial bioactive molecules activate signaling pathways that control host responses and functions [40]. There is now scientific evidence that EVs mediate the effects of microbiota and probiotic strains on host physiology and health [41]. Moreover, they are able to alleviate metabolic, immune and neurological disorders in experimental models of human diseases [41,42,43]. 

Our group has provided new insights into the role of EVs released by the probiotic EcN and commensal *E. coli* strains. We have reported that EVs isolated from gut-resident *E. coli* strains modulate the integrity and repair of the intestinal epithelial barrier at multiple levels [41,44], and elicit immunomodulatory effects on dendritic cells and derived T cell responses. We have shown that EVs are a central mechanism used by the gut microbiota to steadily prime the immune system in a strain-specific manner through mechanisms that involve, at least in part, regulation of miRNAs and immunomodulatory molecules exported through exosomes released by dendritic cells [45,46]. In addition, we have recently reported that nutritional interventions based on the oral administration of EVs from the probiotic EcN or the commensal EcoR12 in neonatal suckling rats stimulate immunity and intestinal maturation in the neonatal period. Pups receiving EVs displayed higher levels of all plasma Ig types and a greater proportion of Tc, NK and NKT cells in the spleen [47]. 

Considering this finding together with previous results showing the ability of EcN EVs to both activate dendritic cells towards a prominent T helper 1 (Th1) response [45] and protect the intestinal epithelial barrier against enteropathogenic *E. coli* (EPEC) infection [48], the present study aimed to explore the effectiveness of interventions involving EVs from the probiotic EcN to protect against RV infection in a neonatal rat model. A parallel intervention with EVs from the commensal EcoR12 was carried out to test for strain-specific effects. We assessed the capacity of probiotic/microbiota EVs to ameliorate diarrhea severity and incidence, and to enhance cellular and specific humoral responses. 

## 2. Results

### 2.1. Evaluation of Body Weight and Morphometric Variables

Infection with RV had no significant impact on animal growth as deciphered by the body weight, body mass index and body height values. The growth parameters were not influenced by the interventions involving EcN EVs or EcoR12 EVs (Figure S1 and Table S1). 

Regarding the relative weight of organs, we centered the analysis on immune-related organs (thymus and spleen) and intestinal tissues (small intestine and cecum) at the active diarrhea period (day 8) and at the end of the study (day 16). Differences between groups were only observed on day 8 (Table 1). RV-infected animals exhibited higher spleen weight values than control animals on day 8. Remarkably, differences were statistically significant in the RV + EV-EcN and RV + EV-EcoR12 groups (*p* < 0.05) and close to significance in the RV group (*p* = 0.08). 

### 2.2. Clinical Evaluation of Diarrhea

**Figure 1:**
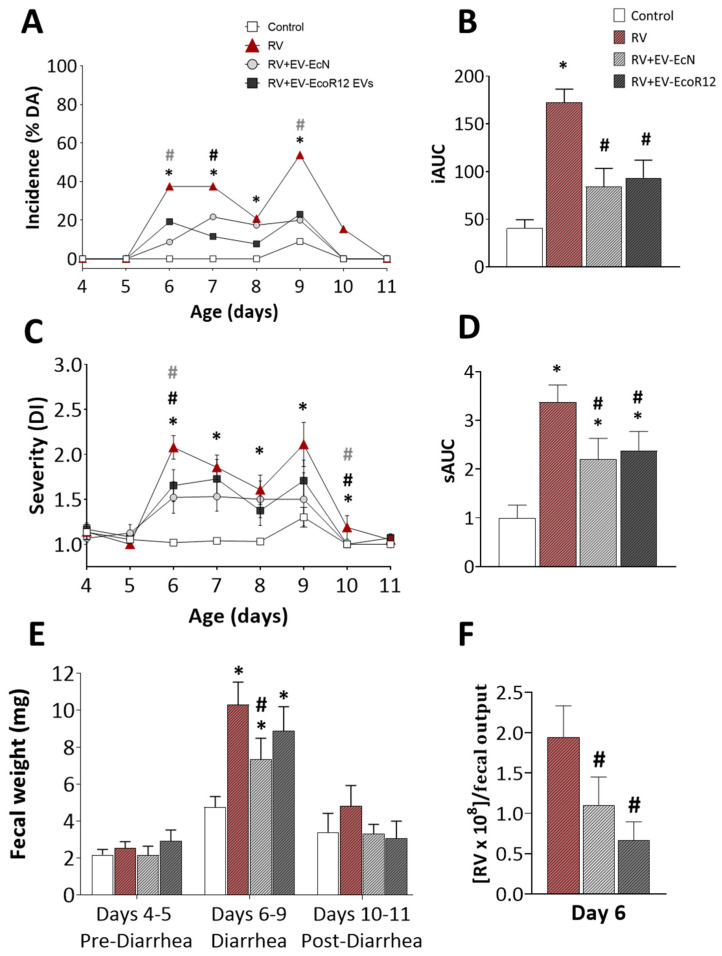
Incidence and severity of RV-induced diarrhea. The clinical variables (**A**) percentage of diarrheic animals (%DA), (**C**) diarrhea index (DI) and (**E**) fecal weight (mg) were evaluated from day 4 (the day before RV inoculation) until day 11 (end of the diarrhea period). (**B**,**D**) The area under the curve (AUC) is shown for the DA and DI graphs (iAUC and sAUC, respectively). (**F**) Viral shedding in feces, expressed as the number of SA-11 particles, referred to the fecal samples pooled on day 6. Viral particles were undetected in feces of control animals. The experimental groups were CON: control group, RV: rotavirus group, RV + EV-EcN: rotavirus group receiving EcN EVs, and RV + EV-EcoR12: rotavirus group receiving EcoR12 EVs. Results are expressed as the mean ± SEM (*n* = 12 animals/group for days 9–11; *n* = 24 animals/group for days 4–8). Statistical differences: * *p* < 0.05 compared to CON, # *p* < 0.05 compared to the RV group (by post hoc Dunn’s multiple comparison test). The Chi squared test was used for statistical analysis of DA% in (**A**).

RV-induced diarrhea was assessed from day 4 (the day before RV inoculation) until day 11 (last day of diarrhea), considering several variables. The incidence, calculated as the percentage of animals displaying diarrhea (% DA), showed that in the RV group, there was a two-peak shape evolution with maximum % DA on days 6 and 7 (40%), and later, on day 9 (50%). On day 11, none of the animals in the RV group showed diarrhea (Figure 1A). Biphasic disease profiles for DA and diarrhea index (DI) values with two pics on days 2 and 4 post-inoculation have already been described for this experimental model [49]. Also, studies in infant mice reported two peaks of RV replication at the same days post-infection [50]. The interventions involving EcN EVs or EcoR12 EVs were able to reduce the proportion of animals with diarrhea with few differences between them. Consistently, the area under the curve (AUC) of the incidence (iAUC) curve over the evaluation period was significantly lower for the interventional groups (RV + EV-EcN and RV + EV-EcoR12) compared to the RV group (Figure 1B). In addition, both interventions reduced other incidence parameters, such as the maximum percentage of diarrheic animals (MDA) and the maximum percentage of diarrheic feces (MDF), a value referred to the total number of fecal samples per day in each group (Table 2). 

The severity of diarrhea was assessed by two approaches: the diarrheic index (DI) and the fecal weight. Inoculation of rotavirus SA-11 resulted in mild diarrhea, with maximum DI values ranging from 2.25 to 3. Both interventional groups, RV + EV-EcN and RV + EV-EcoR12, displayed an overall reduction in DI values compared to the RV group (Figure 1C). For both interventional groups, differences were statistically significant (*p* < 0.05) on day 6 (one day after the RV inoculation). Globally, values of the AUC calculated from the DI plot revealed that both interventions significantly reduced the diarrhea score (*p* < 0.05) (Figure 1D). Improvement of the diarrhea severity in animals receiving EcN or EcoR12 EVs was also evidenced by their maximum diarrhea index (MDI), which weas significantly lower than that of the RV group (Table 2).

The fecal weight was used as an objective variable to characterize the severity of diarrhea (Figure 1E). As expected, no significant differences in the fecal weight output between the CON and RV groups were observed before RV inoculation (days 4–5). The weight of the fecal output during the following four post-infection days, coincident with the acute diarrhea period (days 6–9), was higher in all infected groups compared to the CON animals. However, the intervention with EcN EVs reduced the fecal weight during the acute diarrhea period (*p* < 0.05) compared to the RV group. Similarly, the fecal weight tended to decrease in the RV + EV-EcoR12 group compared to the RV group. In the post-diarrhea period (days 10–11), all groups behaved similarly as diarrhea was already solved.

Regarding the duration of diarrhea, the clinical symptoms started on day 6. The diarrhea period (DP) and the number of days with diarrhea (DwD) were significantly reduced (*p* < 0.05) by the intervention with EcoR12 EVs (Table 2).

### 2.3. Viral Shedding and In Vitro Blocking Assay

Quantification of RV particles in stool samples was carried out on day 6, according to previous reports showing that, in the suckling rat model, the maximum SA-11 clearance occurs on the first day after inoculation [19]. Consistent with these findings, a high number of SA-11 particles in feces was detected in the RV group (Figure 1F). The number of fecal viral particles was significantly reduced by the interventions involving either EcN EVs or EcoR12 EVs (*p* < 0.05).

It was examined whether lower counts of SA-11 particles in the feces of animals administered EcN or EcoR12 EVs could be attributed to an indirect interference caused by vesicle binding to virus particles (Figure S2). Pre-incubation of SA-11 with different concentrations of EVs isolated from EcN or EcoR12 did not cause any blocking effect in virus detection. This finding ruled out any interference with the immunological quantification of SA-11 in feces.

### 2.4. Systemic Humoral and Anti-RV Antibody Response

**Figure 2:**
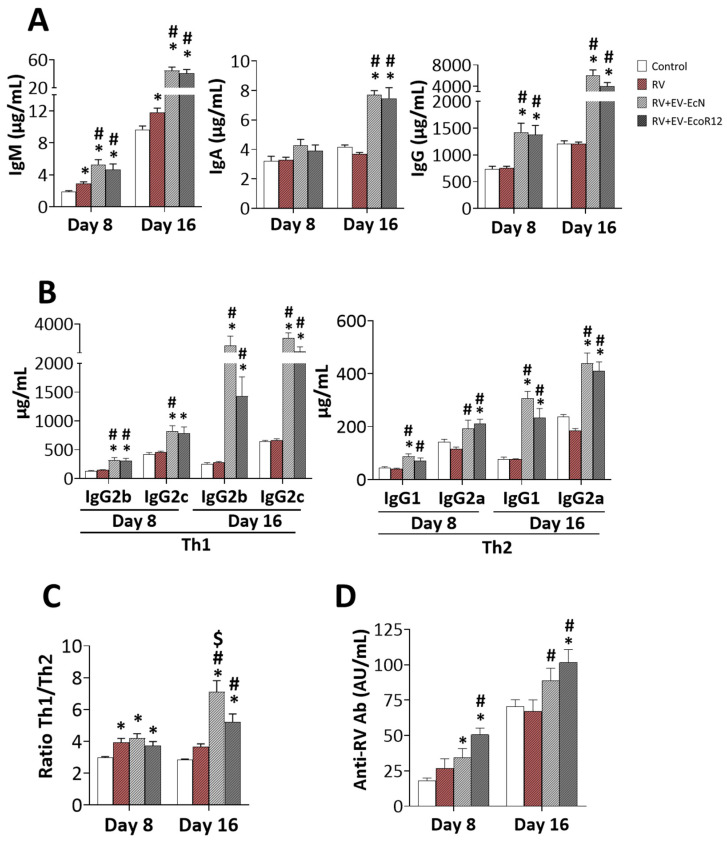
Quantification of anti-RV antibodies and immunoglobulins in plasma on days 8 and 16. The experimental groups are CON: control group, RV: rotavirus group, RV + EV-EcN: rotavirus group receiving EcN EVs, and RV + EV-EcoR12: rotavirus group receiving EcoR12 EVs. (**A**) Concentration of total IgG, IgM and IgA in plasma (µg/mL). (**B**) Concentration of IgG subtypes in plasma (µg/mL). (**C**) The Th1/Th2 Ig ratio refers to the relation (IgG2b + IgG2)/(IgG1 + IgG2a). (**D**) The concentration of specific anti-RV Ab (IgG + IgA + IgM) is given in Arbitrary Units/mL. Results are expressed as ± SEM (*n* = 12 animals/ group on both day 8 and day 16). Statistical differences: * *p* < 0.05 compared to CON, # *p* < 0.05 compared to the RV group, $ *p* < 0.05 between RV-EV-EcN and RV-EV-EcoR12 groups (by post hoc Dunn’s multiple comparison test).

The analysis of the systemic humoral response revealed that RV infection, and specially the interventions with EcN and EcoR12, induced changes in the levels of total circulating Ig (Figure 2A). RV infection affected plasma IgM levels, which were higher than those of the control animals, both on day 8 and day 16 (*p* < 0.05). Although no significant changes in total plasma IgG or IgA levels were observed in the RV group compared to CON, infection with RV increased the Th1/Th2 ratio (*p* < 0.05), estimated from the concentration of the different IgG subtypes (IgG2b + IgG2c/IgG1 + IgG2a) (Figure 2B,C). Minor changes in the levels of IgG subtypes may account for the immune response being biased towards the Th1 phenotype, which is essential to fight against infection. Remarkably, the interventions involving EcN or EcoR12 EV promoted a highly significant increase in the plasma levels of IgM, IgG and all the IgG subtypes compared to the CON and RV groups in both the acute and post-diarrhea periods (days 8 and 16, respectively). Regarding changes in the IgA levels caused by the interventions, differences versus CON and RV were only statistically significant on day 16 (*p* < 0.05) (Figure 2A). 

Administration of EcN or EcoR12 EVs stimulated the humoral immune response to better fight against viral infections, as deciphered from the values of the Th1/Th2 Ig ratio (Figure 2C). On day 8, animals in the RV + EV-EcN and RV + EV-EcoR12 groups exhibited Th1/Th2 Ig ratios similar to those of the RV group, whereas on day 16, the ratio values were significantly higher in both interventional groups (*p* < 0.05 vs. RV group). Particularly, EcN-EV-administered animals displayed greater ratio values than EcoR12-EV-treated animals (*p* < 0.05). Although all the IgG subtypes were increased by the interventions, changes predominantly affected the Th1 isotypes (around an eight-fold increase for IgG2b and a four-fold increase for IgG2c vs. the RV). 

To evaluate the impact of the interventions involving EcN EVs or Ecor12 EVs on the anti-RV humoral response, the plasma concentration of total anti-RV Ab (IgG + IgA + IgM) was measured on day 8 and on day 16 (Figure 2D). On day 8 (active diarrheic period), animals in the RV group exhibited a slight increase (not statistically significant) in the level of circulating anti-RV Ab compared to the CON group. This difference was no longer observed on day 16 (end of the study). Importantly, the interventions with EcN EVs or EcoR12 EVs, although displaying less diarrhea, stimulated the specific anti-RV humoral response. The RV + EV-EcN and RV + EV-EcoR12 groups showed higher levels of anti-RV Ab than the CON and RV groups both on day 8 (*p* < 0.05 vs. CON and RV + EV-EcoR12 also vs. RV) and on day 16 (*p* < 0.05 vs. CON and RV).

### 2.5. Cellular Immune Response

The cellular immune response was assessed by measuring the relative proportion of the main splenic lymphocyte subsets on day 16 by flow cytometry. Representative dot plots for each lymphocyte subtype gate are shown in Figure S3. The results presented in Table 3 showed that neither the infection with SA-11 nor the interventions involving EcN EVs or EcoR12 EVs significantly altered the proportion of total B and T lymphocytes compared to untreated CON animals on that day. However, a tendency towards increased proportion of T cells was observed in the RV + EV-EcN and RV + EV-EcoR12 interventional groups. 

Remarkably, the relative percentage of spleen Tαβ cytotoxic (Tc) cells (TCRαβ+ CD8+ NK-) was increased in all RV-infected groups independently of whether they were treated or not with microbiota EVs (*p* < 0.05 vs. CON). In contrast, the three infected groups showed a minor proportion of Th cells (TCRαβ+ CD4+ NK-). Consistently, the ratio between the spleen Tc/Th cells was higher in the RV-infected groups. 

In RV-infected pups, the interventions with EcN EVs or EcoR12 EVs specifically raised the relative percentage of TCRϒδ+ (*p* < 0.05 vs. CON and RV), particularly in the CD8+ subpopulation. Moreover, the suckling rats in the two interventional groups exhibited higher proportions of NKT CD8+ and NK CD8+ than those in the CON and RV groups. When compared to the RV group, differences were statistically significant for the RV + EV-EcoR12 (*p* < 0.05) group and close to significance for the RV + EV-EcN *(p* < 0.09) group. Importantly, for NK CD8+ cells, differences between RV + EV-EcN and RV were significant (*p* < 0.05) if Dunn’s multiple test was applied to the three infected experimental groups (RV, RV + EV-EcN and RV + EV-EcoR12), a finding that reinforces the influence of EcN EVs on the differentiation of this lymphocyte subpopulation. No significant differences in the proportion of spleen Treg cells were observed between groups. 

### 2.6. Gene Expression Analysis in Small Intestine

**Figure 3:**
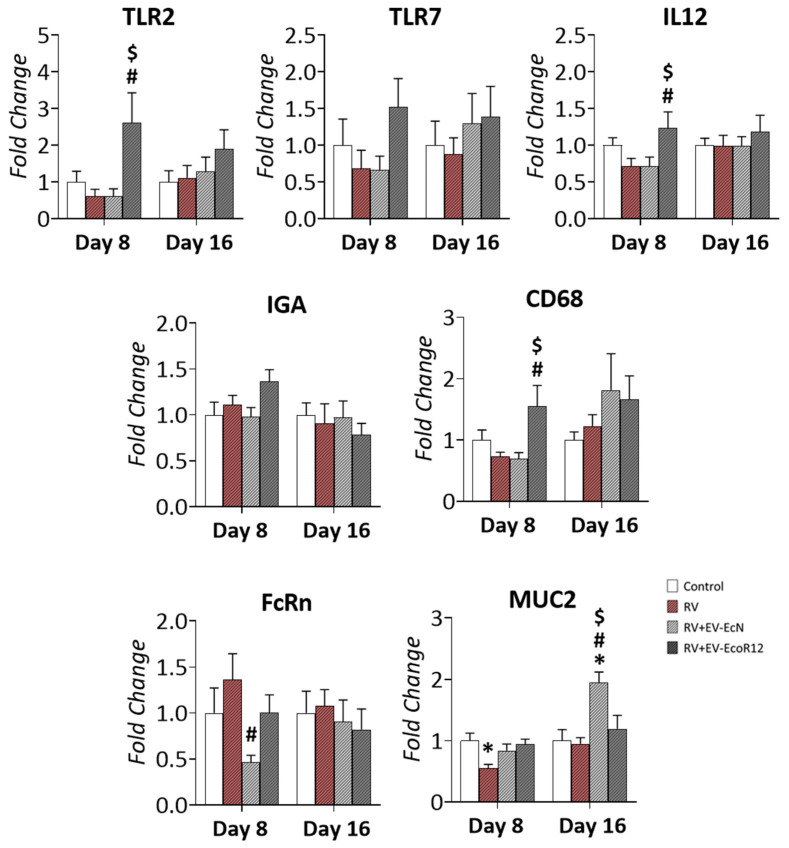
Gene expression analysis in small intestinal tissue collected on days 8 and 16. The experimental groups were CON: control group, RV: rotavirus group, RV + EV-EcN: rotavirus group receiving EcN EVs, and RV + EV-EcoR12: rotavirus group receiving EcoR12 EVs. The transcription levels of the indicated genes were quantified by RT-qPCR in jejunum samples. Relative mRNA levels in the interventional groups were calculated with respect to the CON group (expression value set to 1 at each age). Results are expressed as ± SEM (*n* = 12 animals/group on both day 8 and day 16). Statistical differences: * *p* < 0.05 compared to CON, # *p* < 0.05 compared to the RV group, $ *p* < 0.05 between RV-EV-EcN and RV-EV-EcoR12 groups (by post hoc Dunn’s multiple comparison test). Abbreviations: *TLR*, Toll-like-receptor; *IGA*, immunoglobulin A; *CD68*, cluster of differentiation 68; *FcRn*, neonatal constant fragment receptor; *IL12*, interleukin-12; *MUC2*, mucin 2.

To further examine the effect of the interventions involving EcN and EcoR12 EVs on the intestinal function of RV-infected animals, the expression of several genes involved in host immune responses against infection (*TLR2*, *TLR7*, *IL12*, *IGA* and *CD68*), mucin production (*MUC2*) and intestinal maturation (neonatal constant fragment receptor (*FcRn*)) was measured with reverse transcription–quantitative PCR (RT-qPCR) in the small intestine on days 8 and 16 (Figure 3). 

Infection with SA-11 did not cause significant changes in the expression of the genes analyzed. Nevertheless, the interventions with microbiota EVs modified the expression of some genes in a strain-specific manner. 

Concerning the immune-related genes, administration of EcoR-12 EVs drastically upregulated the expression of genes encoding the immune receptors TLR2 and TLR7 (*p* = 0.095) and the macrophage glycoprotein CD68 on day 8 compared to untreated (CON) or infected (RV) animals (*p* < 0.05). In contrast, pups in the RV + EV-EcN group exhibited *TLR2, TLR7* and *CD68* mRNA levels similar to those of the CON and RV groups. In addition, animals receiving EcoR12 EVs displayed higher *IL12* mRNA levels than RV-infected animals (*p* < 0.05). In the RV and RV + EV-EcN groups, the *IL12* expression levels tended to be lower than the CON group. No differences were observed in the expression of *IGA*, although a tendency to increase was observed in the RV + EV-EcoR12 group compared to CON and RV. Importantly, the changes induced by EcoR12 EVs in immune-related genes were not apparent on day 16, after infection resolution.

The expression of the gene *FcRn*, a marker of intestinal immaturity, tended to be higher in the RV group compared to CON. Importantly, only the intervention with EcN EVs promoted a significant reduction in *FcRn* mRNA levels compared to the RV group (*p* < 0.05). This effect was not caused by the intervention with EcoR12 EVs. In this interventional group, *FcRn* expression levels did not significantly differ from those of the CON group. Moreover, EVs from the probiotic EcN specifically upregulated *MUC2* expression on day 16 (*p* <0.05). On day 8, *MUC2* mRNA levels tended to be reduced in the RV group compared to the CON, but preserved, close to the control levels, in both interventional groups, RV + EV-EcN and RV + EV-EcoR12.

### 2.7. Intestinal Serotonin (5-HT)

**Figure 4:**
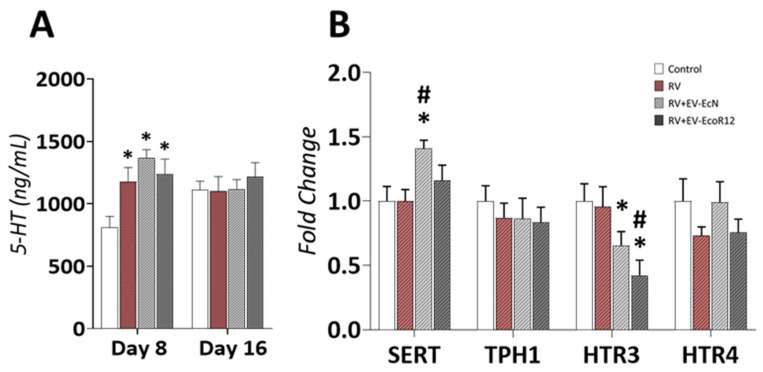
Analysis of serotonin production and regulation by the interventions in RV-infected neonatal rats. The experimental groups were CON: control group, RV: rotavirus group, RV + EV-EcN: rotavirus group receiving EcN EVs, and RV + EV-EcoR12: rotavirus group receiving EcoR12 EVs. (**A**) Serotonin levels in intestinal wash assessed by ELISA. (**B**) Gene expression analysis in small intestinal tissue collected on day 8 (diarrhea period) measured by RT-qPCR. The transcription levels of the indicated genes were quantified by RT-qPCR. Relative mRNA levels in the interventional groups were calculated with respect to the CON group (expression value set to 1). Abbreviations: *THP1*, tryptophan hydroxylase 1; *SERT*, serotonin transporter SLC6A4; *HTR3*, serotonin 5-HT3 receptor; *HTR4*, serotonin 5-HT4 receptor. In both panels, the results are expressed as ±SEM (*n* = 12 animals/group). Statistical differences: * *p* < 0.05 compared to CON, # *p* < 0.05 compared to the RV group (by post hoc Dunn’s multiple comparison test).

It is known that RV infection induces the release of serotonin, an important mediator of RV-associated diarrhea [6]. To analyze the impact of the interventions involving EcN EVs or EcoR12 EVs on intestinal serotonin levels in RV-infected pups, 5-HT was quantified by ELISA in gut wash samples (Figure 4A). 

On day 8, intestinal 5-HT levels were significantly higher in the three RV-infected groups compared to the CON group (*p* < 0.05). Thus, the interventions with EcN or EcoR12 EVs did not prevent RV-induced serotonin release during the acute diarrhea phase. On day 16, all the experimental groups (CON and RV-infected groups) exhibited similar secreted serotonin levels in small intestine-derived samples (Figure 4A).

To further analyze the regulatory mechanisms activated by microbiota EVs, the expression of genes involved in serotonin synthesis (*TPH1*) and transport (*SERT*) was measured with RT-qPCR in small intestinal tissue on day 8 (Figure 4B). No significant differences in the *THP1* mRNA levels were observed between the four experimental groups, whereas *SERT* expression levels were increased in RV-infected pups administered microbiota EVs. Differences were statistically significant for EcN EVs (*p* < 0.05 vs. CON and RV). 

Expression analysis of genes encoding serotonin receptors revealed that the interventions involving EcN EVs or EcoR12 EVs significantly decreased the expression of *HTR3* in RV-infected rats compared to the CON group. The inhibitory effect promoted by EcoR12 EVs was more pronounced, resulting in differences that were statistically significant compared to the RV group. Differences between RV + EV-EcN and RV were close to significant (*p* = 0.08). In contrast, pups in the RV group exhibited *HTR3* mRNA levels that did not statistically differ from those of the CON group (Figure 4B). Concerning the modulation of *HTR4* gene expression, no significant differences were observed between the four experimental groups, although RV and RV + EV-EcoR12 groups displayed a tendency towards lower *HTR4* mRNA levels compared to the CON and RV + EV-EcN groups (Figure 4B).

### 2.8. Histological Analysis of Mucosal Morphology and Goblet Cell Numbers

**Figure 5:**
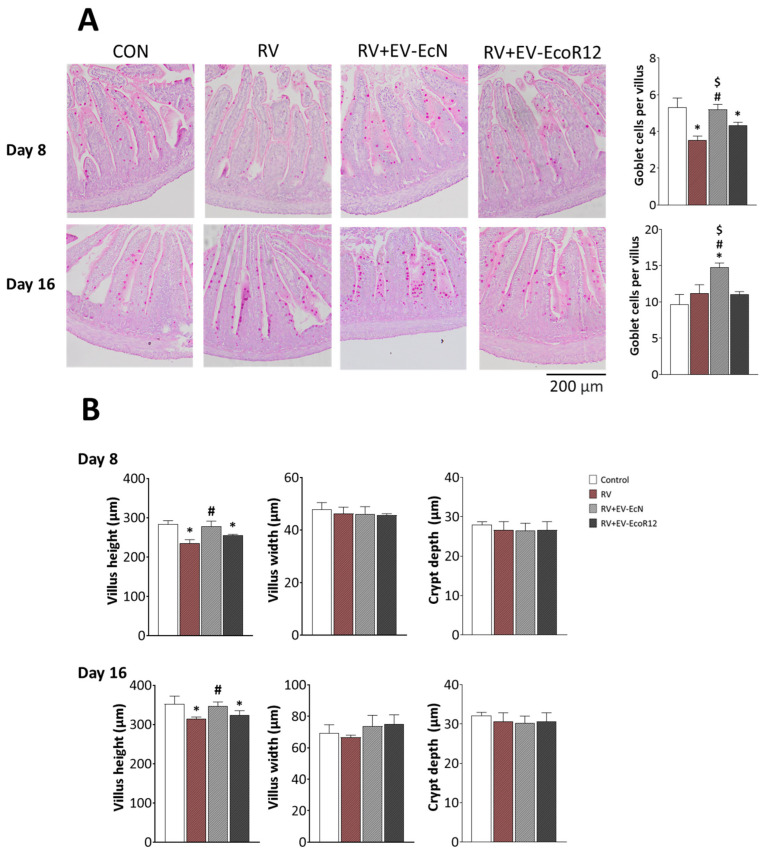
Mucosal morphology of small intestinal tissue collected from all the experimental groups after 3 days of infection (day 8 of life) and at the end of the procedure (day 16). (**A**) Representative images of distal jejunum sections of animals stained with periodic acid Schiff (PAS) and counterstained with hematoxylin (magnification 10×). The number of goblet cells (pink dots) per villus is shown on the right graph at both time points. (**B**) Villi height, width and crypt depth values. Results are expressed as mean ± SEM (*n* = 12/group). Statistical differences: * *p* < 0.05 compared to the control; # *p* < 0.05 compared to the RV group; $ *p* < 0.05 between RV + EV-EcN and RV + EV-EcoR12 groups (by post hoc Bonferroni test).

To assess the influence of EV interventions on the RV-induced morphological alterations in the small intestine, jejunum sections collected on day 8 and day 16 were processed for goblet cell staining (Figure 5A). On day 8 (3 days post-infection), the number of goblet cells in the RV group was significantly lower than the CON group (*p* < 0.05). Only the intervention with EVs from the probiotic EcN was able to prevent the reduction in the number of goblet cells in RV-infected animals, yielding similar control numbers. In contrast, the number of goblet cells in the RV + EV-EcoR12 group was similar to that of RV infected animals. On day 16, when the infection was resolved, the number of goblet cells did not differ between the CON, RV and RV + EV-EcoR12 groups. Importantly, significantly higher goblet cell numbers were enumerated in infected pups treated with EcN EVs. 

Infection with RV also altered villi morphology, specifically the villus length (Figure 5B). In the RV group, villi became significantly shorter compared to the control group (*p* < 0.05) at both time points. Villus width and crypt depth were not affected by RV infection. The intervention involving EcoR12 EVs did not prevent RV-induced villi atrophy. In contrast, treatment with EcN EVs was capable to preserve the normal villus length in infected animals (*p* < 0.05 vs. RV group).

## 3. Discussion

Rotavirus infection is one of the main causes of acute gastroenteritis in children that results in severe diarrhea and loss of fluids. Nowadays, the role of the gut microbiota in preventing and modulating viral infections is widely recognized [51]. Thus, besides proper oral rehydration and dietary interventions, the administration of probiotics has been investigated in clinical trials to lessen diarrhea and gastrointestinal signs [52,53,54,55,56]. In addition, oral RV vaccines have been included in childhood immunization programs to prevent diarrhea and reduce risk of death in low-income countries. However, current vaccines show limited efficacy, especially in children suffering malnutrition and in poor health and hygiene conditions [14,57]. In this context, probiotic intervention represents a potential strategy to enhance immunity and vaccine efficacy, as evidenced by the probiotic EcN in a gnotobiotic piglet model [31]. Also, bacterium-like particles derived from immunobiotic Lactobacilli have been shown to improve the intestinal and systemic immune responses triggered by an attenuated RV vaccine [58]. 

Considering the potential risk of probiotic use in newborns and immunocompromised individuals, the focus on new biotic therapeutic/nutritional strategies is now shifting from viable probiotic bacteria to postbiotics [32,33]. In the present study, EVs form the probiotic EcN and the gut commensal EcoR12 have been analyzed as a postbiotic intervention to ameliorate clinical symptoms and enhance immunity in a preclinical model of early-life RV infection in suckling rats. The dose of EVs was established in accordance with previous studies showing that oral administration of EVs isolated from these microbiota strains in the neonatal period was safe and well tolerated [47]. 

In the present approach, infection with the simian RV strain SA-11 in rat pups on day 5 of life induced moderate diarrhea without any negative impact on body weight or growth variables. This finding was consistent with previous studies using the RV SA-11 infection model in neonatal rats [19,20,49,59,60]. The interventions based on daily administration of EcN EVs or EcoR12 EVs in the first two weeks of life did not affect the animal weight, length or BMI, but significantly improved clinical symptoms by reducing both the severity and the incidence of RV-associated diarrhea. In addition, the intervention with EcoR12 EVs shortened the diarrhea period. To our knowledge, no studies have previously addressed the in vivo effects of microbiota/probiotic EVs on RV infection. In this context, we sought to investigate the mechanisms modulated by the EV interventions to ameliorate diarrhea and improve clinical symptoms.

One of the mediators involved in RV diarrhea is serotonin. It is well established that RV triggers the secretion of serotonin by enterochromaffin cells in the small intestine through the non-structural viral protein (NSP)4 in a Ca^2+^-dependent manner. The released serotonin activates vagal afferent nerves linked to vomiting brain structures and contributes to diarrhea and intestinal motility. These actions are primarily mediated by the 5-HT_3_ receptor [6,8,9]. At the gene expression level, RV promotes downregulation of the gene encoding the serotonin transporter SERT in the ileum of infected mice, without modifying the expression of the gene encoding the rate-limiting enzyme THP1 in the serotonin synthesis pathway [6]. In the neonatal rat model employed in this study, intestinal serotonin levels were also increased in the RV group. However, contrary to what has been described in mice, RV infection did not modify *SERT* expression. This divergence could be attributed, among other factors, to differences in the analysis timing (1–2 days post-infection vs. 3 days post-infection) or the section of the small intestine examined (ileum or jejunum). The interventions with EcN EVs or EcoR12 EVs did not reduce intestinal serotonin levels, although *SERT* mRNA levels were higher in both EV-treated groups compared to the RV group, especially in the RV + EV-EcN group. The serotonin transporter SERT plays an important role in the reuptake and clearance of the serotonin released in the lamina propria, channeling this mediator towards degradation in enterocytes. Reduction/dysfunction of SERT has been observed in enteropathogenic infections [61] and inflammatory bowel disease [7], causing disturbances in intestinal function such as enhanced motility and fluid secretion. Several reports showed that certain probiotics (*Lactobacillus rhamnosus GC*) or gut-beneficial bacteria (*Akkermansia muciniphila* and *Faecalibacterium prausnitzii*) are capable of upregulating *SERT* expression at the intestinal tissue [62,63,64]. In the case of *A. muciniphila*, the EVs had a more prominent regulatory effect than live bacteria [63]. The higher levels of intestinal serotonin in the RV + EV-EcN and RV + EV-EcoR12 groups are consistent with other studies proving the ability of gut bacteria, including the probiotic EcN, to enhance serotonin synthesis and bioavailability in the gut [65,66]. Besides the evaluation of the effects of EcN or EcoR12 EVs on the main genes of serotonin metabolism, we sought to analyze their impact on the gene expression of key serotonin receptors involved in intestinal motility and diarrhea, principally *HTR3* and *HTR4*. Importantly, both EV interventions significantly reduced the expression of *HTR3* in RV-infected pups to levels lower than those of the CON or RV groups. Thus, downregulation of *HTR3* may be one of the mechanisms stimulated by EcN and EcoR12 EVs to alleviate diarrhea in neonatal rats. This conclusion is consistent with the pharmacological action of HTR3 antagonists in improving diarrhea and intestinal inflammation [7]. Regarding *HTR4*, although no statistically significant differences were observed between groups, its expression tended to be reduced in the RV group, whereas in the RV-EV-EcN group, *HTR4* mRNA levels were similar to those of the CON group. The commensal *Bifidobacterium dentium* stimulates production of serotonin by enterochromaffin cells and the released serotonin was shown to activate HTR4 in goblet cells to promote the secretion of MUC2 and TFF3, which contribute to epithelial repair [67]. In accordance with this finding, HTR4 agonists alleviate disease severity and reduce inflammation in models of inflammatory bowel disease [7]. Preservation of *HTR4* expression by EcN EVs in RV-infected neonatal rats may help to preserve intestinal epithelium function and repair, thereby mitigating RV-induced dysfunction. 

The amelioration of diarrhea observed in the EV-treated groups was accompanied by a decrease in the number of viral particles in fecal samples on day 6 of life (one day post-inoculation). This corresponds to the day with the highest viral elimination [19,59]. The high viral load in feces reflects both the elimination of the viral inoculum and the new viruses produced by replication in infected epithelial cells. Several probiotic strains of the *Lactobacillus*, *Bifidobacterium* and *Escherichia* genera have been shown to reduce viral shedding in RV infection models through several mechanisms, including virus sequestering, interference with RV adhesion to target epithelial cells and activation of antiviral humoral and cell immune responses [18,20,24]. In the case of the probiotic EcN, reduced viral shedding titers in gnotobiotic piglets have been correlated with the ability of this probiotic to bind RV particles, to enhance NK cell function and to regulate intestinal and serum IgA response [18,22,29]. In our study, the interventions with EVs did not involve live bacteria. Therefore, this study can provide new insights into establishing the probiotic beneficial effects that are mediated by EVs. In this context, in vitro studies carried out in polarized human colonic cell lines showed that pre-treatment with EcN culture supernatants reduced RV replication similarly to the live probiotic, although the secreted effector molecules with anti-RV inhibitory properties were not elucidated [68]. Here, we show that the released EVs isolated from culture supernatants act as carriers of such biologically active molecules, not only in the case of the probiotic EcN but also for the commensal EcoR12.

Regarding the antibody response, the level of total anti-RV Ab in the RV group did not significantly increase compared to the CON levels. This finding was previously described in a suckling rat model and attributed to the immaturity of the immune system of neonatal rats [59]. However, pups receiving either EcN EVs or EcoR12 EVs showed higher titers of anti-RV Ab in plasma both at day 8 and 16. During the diarrhea period, reduced RV fecal shedding titers in the RV + EV-EcN and RV + EV-EcoR12 groups correlated with increased levels of serum anti-RV Ab. In addition, both EV interventions also improved the overall systemic humoral response. Administration of either EcN or EcoR12 EVs promoted a significant increase in the plasma levels of all tested Ig types (IgA, IgM and IgG) compared to the CON and the RV groups. For IgM and IgG, this effect was observed at days 8 and 16, whereas the IgA response was only evident at day 16. These results are in line with the immunogenic properties of EVs from EcN and EcoR12 reported previously in healthy neonatal rats following a 16-day intervention period [47], and evidenced that a viral challenge can accelerate the vesicle-induced response as early as day 8. Based on the relationship between the different IgG subtypes (Th1/Th2 ratio), RV inoculation induced a shift towards the Th1 response (on day 8) as an attempt to control infection. The Th1/Th2 ratio returned to normal values once the infection was solved (day 16). The early response was maintained in animals receiving EcN or EcoR12 EVs on day 8 compared to the RV group, and significantly increased on day 16. This finding together with the higher titers of anti-RV Ab and Ig in pups of the RV + EV-EcN and RV-EV-EcoR12 interventional groups indicate that EVs from these microbiota strains enhance the humoral response in RV-infected animals. Moreover, since high levels of anti-RV IgA and IgG in serum after infection resolution are good indicators of protection [11,12,69], it can be hypothesized that these interventions based on microbiota EVs can confer protection against RV infections later in life. 

The interventions also enhanced cellular innate immunity. Independently of the administration of EVs, infection with RV raised the proportion of splenic T cytotoxic cells (TRCαβ+, CD8+) compared to uninfected animals. In addition, the interventions involving EVs specifically increased the proportion of NK+ CD8+ and TRCϒδ+ CD8+ cells, this last one being a T cell subset that is considered a primitive immune cell lineage which rapidly drives innate responses against pathogens and other injuries [70]. In the case of the probiotic EcN, the results presented here provide evidence that the enhancement of innate and adaptative immune responses reported in several experimental models of RV infection [18,22,29,31] can be mediated by the released EVs.

Overall, the results related to the modulation of humoral and cellular immunity support the potential of EVs from the probiotic EcN or the commensal EcoR12 to be used as vaccine adjuvants in anti-RV immunization processes involving oral vaccines. There is a need to improve the efficacy of current RV vaccines, especially in low-income countries [57]. The suggested application could yield promising results as it has been described for other vaccination models using a combination of antigens and bacterial EVs [71]. 

Concerning the impact of EVs on the expression of immune-related genes in the small intestine during the diarrhea period (day 8), the results revealed that interventions based on EcoR12 EVs may confer protection against RV by upregulating genes encoding the immune receptor TLR2 and the pro-inflammatory cytokine IL-12. Activation of TLR2 signaling results in NF-κB activation and the subsequent expression of pro-inflammatory cytokines. In the presence of specific viral ligands, this receptor can traffic to endosomes in inflammatory monocytes and activate production of antiviral effectors such as type I interferons [72]. Moreover, IL-12 is a pro-inflammatory cytokine produced and released by innate immune cells that plays a key role in the activation of host antiviral immunity and defense mechanisms for virus elimination [73]. The intervention with EcoR12 EVs also promoted upregulation of the glycoprotein CD68, a marker of macrophage recruitment to the infected sites [74]. In contrast, the intervention involving EcN EVs did not activate the expression of these genes involved in mechanisms for virus eradication. Another gene associated with the adaptative immunity that reflects the maturation state of the neonatal intestine is *FcRn*, which encodes the receptor for the uptake of maternal IgG [75]. Animals in the RV group exhibited a trend toward higher levels of *FcRn* mRNA on day 8 compared to the control group. This observation suggests that RV infection in early life may adversely impact intestine maturation by hindering the progressive reduction in FcRn during suckling. Importantly, the intervention with EcN EVs, but not with EcoR12 EVs, significantly reduced *FcRn* expression on day 8. This finding was consistent with previous studies showing the modulation of *FcRn* by EcN EVs [47], and indicates that vesicles from this probiotic can protect and accelerate intestinal maturation both under health and infection conditions. On day 16, as the infection was eradicated and the small intestine completed its maturation process, *FcRn* expression became similar in all groups. The benefits of EcN EVs on intestinal maturation were also evidenced by their ability to protect against RV-induced villi atrophy by preserving villi length to values similar to those of non-infected control pups. Again, this effect was not observed in the RV + EV-EcoR12 group. In the jejunum of RV-infected mice, villus shortening and atrophy together with loss of mature enterocytes have been associated with a defective absorptive function, which contribute to RV pathogenesis [76]. The results presented here prove that the beneficial effects of interventions based on microbiota/probiotic EVs on intestinal maturation and absorptive functions altered by RV infection are strain-specific. 

Goblet cells and the secreted mucins also play a role as active defense mechanisms against RV infection [50,77]. In response to infection, goblet cells release MUC2 and other proteins, enhancing the mucus barrier as a mechanism to trap virus particles. However, a balanced response is needed as goblet cell exhaustion could result in inflammation [78]. Our study shows that during the acute phase of diarrhea (3 days post-infection), animals in the RV group displayed lower *MUC2* gene expression and number of goblet cells per villus in jejunum samples compared to the CON group. These results were in accordance with those reported in RV infected mice at 2–4 days post-infection [50]. The intervention with EcoR12 EVs did not prevent RV-induced early loss of goblet cells, whereas treatment with EVs from the probiotic EcN was able to preserve *MUC2* expression and the number of goblet cells to values that were indistinguishable from those of uninfected pups from the CON group. After diarrhea resolution (day 16), mucin expression and goblet cell numbers in the RV and RV + EV-EcoR12 groups were normalized to CON values. In contrast, animals receiving EcN EVs displayed significantly higher *MUC2* mRNA levels and goblet cells in jejunum samples in the post-diarrhea period than the other groups. In this interventional group, the increased expression of *MUC2* on day 16 is consistent with the higher number of goblet cells. Therefore, interventions based on EVs from the probiotic EcN can counteract the RV-induced alteration of goblet cell function by preserving mucus production and barrier function, both during the diarrhea period and after infection resolution.

This study provides evidence that microbiota EVs are a safe postbiotic alternative to the utilization of live probiotic bacteria in ameliorating RV clinical symptoms and enhancing innate and adaptative immune responses in the neonatal period. Importantly, the beneficial effects of interventions involving microbiota/probiotic EVs are strain-specific. In addition, this study suggests the healing potential of interventions involving EcN or EcoR12 EVs in protecting newborns against other possible enteric infections. This is especially relevant in the case of preterm newborns, who are highly susceptible to necrotizing enterocolitis (among other infections) due to the functional immaturity of the intestinal system [79]. Clinical trials are needed to prove the benefits of microbiota EV-based interventions in human infants. Moreover, the ability of EcN or EcoR12 EVs to enhance humoral and cell immune responses points to their potential application as adjuvants to boost the immunogenicity and protective efficacy of anti-RV vaccines. In comparison with studies analyzing the efficacy of the probiotic EcN as a vaccine adjuvant in gnotobiotic piglets [31], our experimental model evidences the efficacy of interventions involving the isolated EVs in improving infection symptoms and immunity in the presence of indigenous microbiota.

## 4. Materials and Methods

### 4.1. Bacterial Strains and Isolation of Extracellular Vesicles

EVs were obtained from two intestinal *Escherichia coli* isolates: the probiotic EcN (Ardeypharm, GmbH, Herdecke, Germany) and the commensal EcoR12 that is included in the ECOR reference collection [80]. The strains were grown in Luria–Bertani broth (LB). 

Bacterial EVs were isolated from the culture supernatants as reported previously [46]. Briefly, the cell-free supernatants obtained after centrifugation of the bacterial cultures were filtered through 0.22 filters (Merck, Millipore, Burlington, MA, USA) to eliminate residual bacteria and then concentrated using Centricon Plus-70 100 kDa filters (Merck, Millipore, Burlington, MA, USA). EVs were then collected using ultracentrifugation at 150,000× *g* for 1 h at 4 °C, washed twice in phosphate-buffered saline (PBS) and finally resuspended in PBS. The EV samples were tested for sterility using LB plates and quantified in terms of protein concentration with the Pierce BCA protein assay. Samples were also quantified using the lipophilic fluorescent dye FM4-64 (Thermo Fisher Scientific, Barcelona, Spain) that intercalates into the vesicle membrane. This assay allowed us to verify that equal amounts of EcN EVs and EcoR12 EVs were being used in the experimental protocol [47]. For the FM4-64 assay, several dilutions of the stock EV samples (40 μg/mL) were incubated with FM4-64 (5 μg/mL in PBS) for 10 min at room temperature. Reactions containing only EVs or the FM4-64 probe were processed as negative controls. After excitation at 515 nm, emissions at 640 nm were measured with the modular multimode plate reader Varioskan Lux (Thermo Fisher Scientific, Barcelona, Spain). Samples with equal concentration of EVs, expressed as µg protein/mL, yielded similar fluorescence intensity values. EV samples were aliquoted and stored at −20 °C until use.

### 4.2. Virus

The simian rotavirus strain SA-11 was produced by the “Enteric Virus Group-UB” at the University of Barcelona. Rotavirus was propagated in fetal African green monkey kidney cells (MA-104) and titrated as TCID_50_/mL (TCID, tissue culture infection dose) [49]. 

### 4.3. Animals

Animal procedures were approved by the Ethical Committees for Animal Experimentation (CEEA) of the UB and the Catalonian Government (CEEA-UB Ref.169/20 and Ref.11461, respectively) in compliance with the EU-Directive 2010/63/EU. Twelve G14 pregnant Lewis rats were purchased from Janvier (Le Genest-St-Isle, France) and were individually housed in cages (2184 L Eurostandard Type II L, Tecniplast, West Chester, PA, USA) containing tissue paper and fibrous particle bedding. Rats were monitored daily until natural delivery. The day of birth was assigned as day 1 of life. The next day, litters were randomly distributed into 4 experimental groups with 3 lactating dams each. The number of pups was unified to 8 per dam, maintaining a comparable proportion (40–60%) of males and females per litter. The rats were housed in controlled conditions of temperature, humidity and light/dark cycles of 12 h in an isolated room authorized for work in Biosafety Level 2, at the Animal Experimentation Unit (UEA) of the Diagonal Campus at the University of Barcelona (UB). Dams received a commercial AIN-93 G diet (Teklad Global Diet 2014, INOTIV, Indianapolis, IN, USA) [81] and water ad libitum. Neonatal rats had free access to maternal milk. 

### 4.4. Experimental Design

**Figure 6:**
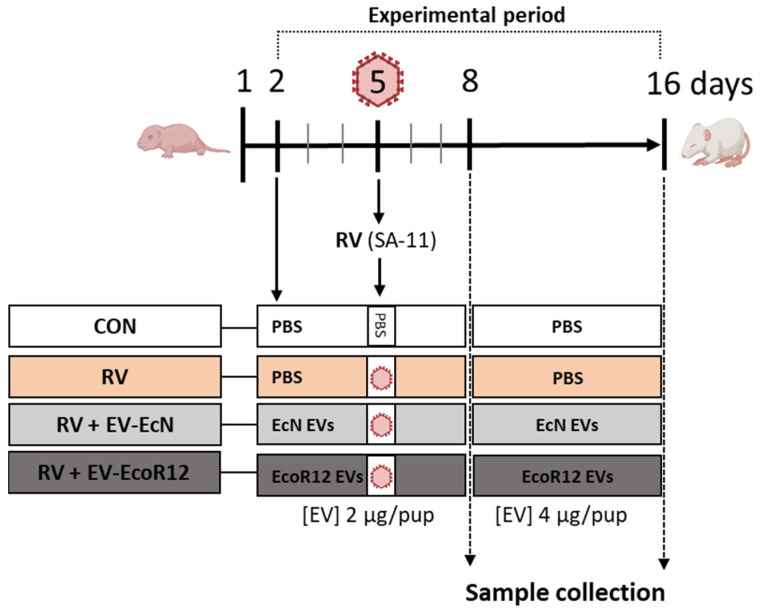
Experimental design. Days are numbered according to the day of birth (day 1). The four experimental groups are CON: non-infected, non-treated animals; RV: animals infected with SA-11; RV + EV-EcN: animals infected with SA-11 and receiving EVs from the probiotic EcN; and RV + EV-EcoR12: animals infected with SA-11 and receiving EVs from the commensal EcoR12. Rotavirus SA-11 was inoculated on day 5 in all experimental groups except in the control. In the interventional groups, pups were intragastrically administered with EVs from day 2 until the end of the protocol at the indicated doses. The other groups (CON and RV) were given the same volume of the vehicle (PBS).

Litters were assigned to different experimental groups: control (CON), rotavirus infection (RV), RV-infected animals receiving EVs from EcN (RV + EV-EcN) and RV-infected animals receiving EVs from EcoR12 (RV + EV-EcoR12). Each experimental group consisted of 3 litters with 8 pups each (*n* = 24/group). The experimental design is shown in Figure 6.

Neonatal rats were administered EcN or EcoR12 EVs by oral gavage from the second (day 2) to the sixteenth day of life (day 16). The selected dose of EVs was based on previous studies [47]. Considering the increase in body weight, the EV groups received a concentration of EVs of 2 µg protein/animal/day until day 8. From day 9, the dose was increased to 4 µg protein/animal/day and maintained until the last day of the procedure. The administered volume was adjusted to 100 µL. The CON group received an equal volume of the vehicle (PBS). On day 5 of life, rotavirus SA-11 was inoculated to the suckling rats of the RV, RV + EV-EcN and RV + EV-EcoR12 groups by oral gavage (2 × 10^8^ TCID_50_ RV/rat) in 100 µL of PBS) as previously described [49]. The CON group received the PBS vehicle instead. For animal handling, the mother was separated to a new cage and the pups were kept in the home cage. RV was inoculated 30 min after the administration of the EVs or PBS. Pups were brought together with the corresponding dams after a 45 min period in order to avoid interference between the RV and milk components. On day 8 of life (three days after RV inoculation), half of each litter (4 random pups with a similar proportion of males and females) were euthanized to obtain blood and tissue samples (3 litters/group, *n* = 12). The remaining pups were euthanized at the end of the intervention (day 16 of life).

### 4.5. Clinical Evaluation and Fecal Specimen Collection

Body weight was recorded daily from day 2 of life until the end of the intervention. Fecal samples were obtained once daily (from days 4 to 11) by gently pressing and massaging the abdomen. To assess the severity of diarrhea, fecal specimens were weighed and scored in a blinded manner based on their color, texture and amount to obtain a diarrhea index (DI) as described previously [60]. The scores were (1) normal feces, (2) soft yellow feces, (3) loose yellow-green feces and (4) high amount of watery feces. DI values ≥ 2 indicate diarrhea. Incidence of diarrhea was calculated as the percentage of diarrheic animals (% DA, referred to the number of animals displaying scores of DI ≥ 2 in each group) and by the percentage of diarrheic feces (% DF, which considers the number of total samples collected per day in each group). The area under the curve was calculated for the DA and DI graphs (iAUC and sAUC, respectively) as a global value of the process [19]. Fecal specimens were frozen at −20 °C for further analysis. 

### 4.6. Sample Collection

At the end of the experimental procedure (day 8 or day 16 of life), pups were anesthetized with intramuscular injection of ketamine/xylazine (Imalgene 100 mg/mL, Merial Laboratorios, Barcelona, Spain, and Rompun^®^ 20 mg/mL, Bayer Hispania, Sant Joan Despí, Spain). Then, the naso-anal and tail lengths were measured. The body mass index (BMI) was calculated as body weight/length^2^ (g/cm^2^) and the Lee index as 3√weight/length × 1000 (3√g/cm). Once the animals were fully unconscious, euthanasia was performed by opening the peritoneal cavity and disrupting the diaphragm. Blood was collected via cardiac puncture to obtain plasma samples, which were stored at −20 °C. Several digestive and immune-related organs were obtained and weighted to assess morphogenic variables. The length of the small intestine was also recorded. A central portion (1 cm) of the small intestine was cut and placed immediately in liquid nitrogen. Intestinal tissue samples were stored at −80 °C for gene expression analysis. In addition, a fragment of the distal jejunum was collected and processed for histological analysis. The gut wash was obtained as described previously and stored at −80 °C for further analysis [59]. The spleens were processed for lymphocyte isolation as described below.

### 4.7. Fecal Viral Shedding

Fecal samples collected on day 6 were diluted in PBS (10 mg/mL) and homogenized using a FastPrep (MP Biomedicals, Santa Ana, CA, USA). The homogenates were centrifuged (19,000× *g*, 3 min) and the supernatants were frozen at −20 °C until use. SA11 virus particles were quantified by ELISA using 96-well plates (Nunc MaxiSorp, Wiesbaden, Germany) coated with anti-p42 Ab (Meridian Life Science, Memphis, TN, USA), as previously described [20]. Serial dilutions of inactivated SA11 virus particles (ranging from 2.3 × 10^8^ to 1.8 × 10^6^/mL) were used as the standard curve.

### 4.8. In Vitro Blocking Assay

To test the ability of EVs to bind the SA-11 particles, an in-house in vitro blocking assay was performed as previously described [19]. Briefly, RV was diluted in PBS-Tween at a final concentration of 5.8 × 10^7^/mL. Starting from the concentration administered to the neonatal rats, different dilutions of EVs were prepared to obtain concentrations ranging from 40 µg/mL to 1.25 µg/mL, and pre-incubated with the virus (1:1) for 30 min. Then, free, noncoated viral particles were quantified by ELISA as described above (fecal SA-11 shedding). 

### 4.9. Quantification of Total Immunoglobulins and Specific Anti-Rotavirus Antibodies in Plasma

Plasma concentration of IgA, IgM and IgG isotypes (IgG1, IgG2a, IgG2b, IgG2c) were quantified using ProcartaPlex™ Multiplex immunoassay (Thermo Fisher Scientific, Barcelona, Spain), as described in previous studies [47,59]. Briefly, specific color-coded capture beads were bound to the Ig of interest. After adding the different detection antibodies (Abs) conjugated to phycoerythrin (PE), the concentration of each analyte was obtained using the MAGPIX^®^ analyzer (Luminex Corporation, Austin, TX, USA) at the Cytometry Service of the Scientific and Technological Centers of the University of Barcelona (CCiT-UB).

Specific anti-RV Abs (IgA + IgG + IgM) in plasma were quantified by ELISA as reported in previous studies [20]. Briefly, 96-well plates were coated with UV-inactivated SA11 particles (10^5^/mL), blocked with PBS-1% BSA for 1 h and incubated with dilutions of sera during 3 h. All the steps were carried out at room temperature. After washing, peroxidase-conjugated polyclonal anti-rat Ig (Dako, Barcelona, Spain) was added and incubated with the substrate following the manufacturer’s instructions. The standard used was a pooled serum from RV-inoculated rats available and standardized in the laboratory. The absorbance was measured using a microplate photometer (LabSystem Multiskan, LabX) and analyzed with the ASCENT version 2.6 software (Thermo Fisher Scientific, Barcelona, Spain). 

### 4.10. Lymphocyte Isolation from Spleen and Immunophenotyping by Flow Cytometry 

Spleens were mechanically disaggregated using a sterile 40 μm mesh cell strainer (Thermo Fisher Scientific, S.L.U, Barcelona, Spain) as previously described [82]. Erythrocytes were removed with osmotic lysis and lymphocytes were suspended in Roswell Park Memorial Institute medium (RPMI) supplemented with 10% heat-inactivated fetal bovine serum (FBS), 100 IU/mL streptomycin–penicillin and 2 mM L-glutamine. After staining dead cells with Trypan Blue, the cell numbers and viability were measured using a Countess^TM^ Automated Cell Counter (Invitrogen^TM^, Thermo Fisher Scientific, S.L.U, Barcelona, Spain). 

The phenotype of isolated lymphocytes was analyzed using specific anti-rat monoclonal Abs conjugated to different fluorochromes as previously described [47,82]. The Abs used were fluorescein isothiocyanate (FITC)-TCRαβ, brilliant violet 786 (Bv786)-CD8β, FITC-CD25, PE-CD161a, brilliant violet 605 (Bv605)-TCRγδ, PE-CD4, peridinin chlorophyll protein (PerCP)-CD8α, allophycocyanin (APC)-CD4 brilliant violet 421 (BV421)-CD45RA (all from BD Biosciences) and APC-FoxP3 (eBioscience, Frankfurt, Germany). Briefly, lymphocytes were incubated with saturating concentrations of fluorochrome-conjugated Ab, fixed with 0.5% p-formaldehyde and stored (4 °C, in darkness) until flow cytometry analysis. For Treg analysis, lymphocytes were incubated with anti-CD4 (PE) and anti-CD25 (FITC) Ab (20 min, 4 °C, in darkness), treated with a fixation–permeabilization solution (eBioscience, Frankfurt, Germany) (30 min, 4 °C, in darkness) and then incubated with APC-conjugated anti-Foxp3 Ab (30 min, 4 °C, in darkness) as previously described [45]. A negative control staining with an isotype-matched monoclonal Ab was included in each cell sample. 

Analyses were carried out with an AURORA^TM^ Cytometer (Beckman Coulter, Miami, FL, United States) in the Flow Cytometry Unit of the Scientific and Technological Centers of the UB (CCiT-UB), and data were analyzed with Flowjo v10 software (Tree Star, Inc., Ashland, OR, USA). The results are expressed as percentage of positive cells in in a particular lymphocyte population selected according to their forward-scatter characteristics (FSC) and side-scatter characteristics (SSC) (Figure S3).

### 4.11. Gene Expression Analysis by Reverse Transcription–quantitative PCR (RT-qPCR)

Fragments of the small intestine tissue were homogenized in lysing matrix tubes (MP Biomedicals, Illrich, France) using Fast-Prep-24 equipment (MP Biomedicals, Illrich, France). RNA was isolated with the RNeasy^®^ Mini Kit (Qiagen, Madrid, Spain). RNA concentration and purity were assessed using the Thermo Scientific Varioscan Lux modular multimode reader (Thermo Fisher Scientific, Barcelona, Spain). Synthesis of cDNA was carried out using the TaqMan^®^ Reverse Transcription Reagents (Applied Biosystems, AB, Weiterstadt, Germany) following the manufacturer’s protocol. 

Quantitative PCR was performed using ABI Prism 7900 HT equipment (Life Technologies, Madrid, Spain). The specific PCR TaqMan^®^ primers (AB) were *TLR2* (Rn02133647_s1), *TLR-7* (Rn01771083_s1), *IGA* (Rn01511082_m1), *CD68* (Rn00566655_m1), *FcRn* (Rn00583712_m1), *MUC2* (Rn01498206_m1), *IL12* (Rn00584538_m1), *SERT1* (Rn00564737_m1), *TPH1* (Rn01476867_m1), *HTR3a* (Rn00667026_m1) and *HTR4* (Rn00563402_m1). The endogenous housekeeping gene *Gusb* (Rn00566655_m1) was used as the internal control gene. The relative gene expression was expressed as fold change compared to the CON group and calculated by means of the 2^−ΔΔCt^ formula [83].

### 4.12. Serotonin Quantification by ELISA

Serotonin levels were quantified in gut wash samples with a commercial 96-well Rat Serotonin ELISA kit (Cat. No *MBS725497*, MYBioSource, San Diego, CA, USA) following the recommendations and procedures indicated by the manufacturer (sensitivity 1 ng/mL).

### 4.13. Histomorphometry Analysis/Mucin Staining

Paraffin-embedded tissues from distal jejunum fragments were cut into 6 µm sections and processed for histochemical Periodic Acid Schiff (PAS) staining. The sections were deparaffinized with xylene (Merck) and rehydrated in graded ethanol solutions as described elsewhere [76]. After PAS staining (15 min), slides were washed and counterstained with hematoxylin for 30 s. Images were captured by bright-field microscopy (Olympus BX41, Olympus Corporation, Tokyo, Japan) at 100× magnification. At least 10 villi per animal were recorded and analyzed with ImageJv1.53t. The number of goblet cells were counted in ten well-defined villi from each animal.

### 4.14. Statistical Analysis

Statistical analysis was performed using the Statistical Package for Social Sciences (SPSS v22.0) (IBM, Chicago, IL, USA). Data were tested for normal distribution (by Shapiro–Wilk) and variance equality (by Levene’s test). A conventional one-way ANOVA test was carried out followed by the post hoc Bonferroni when data were homogeneous and had a normal distribution, considering the experimental group as the independent variable. The non-parametric Kruskal–Wallis test followed by the post hoc Dunn’s multiple comparison test was applied for the analysis of non-parametric variables (such as feces scoring) or when data displayed non-normal distribution or dissimilar variances. Differences were considered significant at *p* < 0.05. The results were expressed as mean ± standard error of the mean (SEM).

## 5. Conclusions

This study provides evidence that the beneficial effects of interventions involving microbiota/probiotic EVs are strain-specific. EVs from both the probiotic EcN and the commensal EcoR12 have a preventive effect in RV-infected neonatal rats by different mechanisms. Both interventions potentiate the humoral (Ig) and cellular (NK, Tc and TCRγδ cells) immunity against viral infections to similar levels. In addition, EcoR12 EVs mainly activate macrophage recruitment to the infected sites (*CD68* expression) and TLR signaling towards increased production of pro-inflammatory IL-12, whereas EcN EVs improve intestinal maturation (*FcnR* expression), absorptive function (villus length) and barrier properties (goblet cell numbers and *MUC2* expression). Concerning serotonin modulation, EVs from both strains can alleviate diarrhea by downregulating the expression of the intestinal receptor HTR3. 

Our results suggest that microbiota EVs are a safe postbiotic alternative to the utilization of live probiotic bacteria in ameliorating RV clinical symptoms and enhancing innate and adaptative immune responses in the neonatal period. Moreover, EVs isolated from beneficial Gram-negative microbiota/probiotic strains could be used as adjuvants to enhance the immunogenicity and protective efficacy of anti-RV vaccines and prevent RV outbreaks in developing countries.

## Figures and Tables

**Table 1:** Morphometric variables of intestinal and immune-related organs for the indicated experimental groups. Data corresponding to relative organ weight (g) or length (cm) are presented as percentage of animal body weight (%). The groups were: CON: control group, RV: rotavirus group, RV + EV-EcN: rotavirus group receiving EcN EVs, and RV + EV-EcoR12: rotavirus group receiving EcoR12 EVs.

Day 8	CON	RV	RV + EV-EcN	RV + EV-EcoR12
Intestine weight	3.19 ± 0.06	3.43 ± 0.06	3.41 ± 0.05	3.57 ± 0.11
Intestine length	231.57 ± 7.39	210.64 ± 6.5	226.35 ± 6.42	231.95 ± 10.04
Cecum weight	0.19 ± 0.04	0.20 ± 0.04	0.21 ± 0.04	0.21 ± 0.04
Thymus weight	0.29 ± 0.01	0.30 ± 0.01	0.33 ± 0.03	0.29 ± 0.02
Spleen weight	0.48 ± 0.02	0.59 ± 0.02	0.61 ± 0.04 *	0.60 ± 0.03 *
**Day 16**	**CON**	**RV**	**RV + EV-EcN**	**Rv + EV-EcoR12**
Intestine weight	2.94 ± 0.04	3.03 ± 0.05	3.10± 0.08	2.97 ± 0.05
Intestine length	126.08 ± 5.51	119.74 ± 3.04	128.97 ± 7.26	134.07 ± 4.31
Cecum weight	0.21 ± 0.01	0.21 ± 0.01	0.25 ± 0.02	0.24 ± 0.02
Thymus weight	0.52 ± 0.01	0.50 ± 0.01	0.51 ± 0.02	0.51 ± 0.02
Spleen weight	0.44 ± 0.02	0.50 ± 0.03	0.47 ± 0.03	0.52 ± 0.03

Results are expressed as mean ± SEM (*n* = 12 animals/group). * *p* < 0.05 compared to CON (by post hoc Dunn’s multiple comparison test).

**Table 2:** Clinical variables of the diarrhea process from day 4 to day 11 of life. Results are expressed as mean ± SEM (*n* = 12–24 animals/group). Statistical differences: # *p* < 0.05 compared to the RV group (by post hoc Dunn’s multiple comparison test). The Chi squared test was used for statistical analysis of the incidence variables MDA and MDF.

Clinical Outcome	Variable ^1^	RV	RV + EV-EcN	RV + EV-EcoR12
Incidence	MDA	53.85	21.74	23.08
Incidence	MDF	77.77	36.36	50.00
Severity	MDI	2.15 ± 0.12	1.71 ± 0.16 #	1.76 ± 0.15 #
Duration	DP	2.06 ± 0.28	1.75 ± 0.41	1.25 ± 0.25 #
Duration	DwD	1.88 ± 0.22	1.63 ± 0.32	1.10 ± 0.08 #

^1^ MDA, maximum percentage of diarrheic animals; MDF, maximum percentage of diarrheic feces; MDI, maximum diarrhea index; DP, diarrhea period; DwD, days with diarrhea.

**Table 3:** Proportion of spleen lymphocytes on day 16 assessed by flow cytometry analysis. The main populations are numbered (1–6) and highlighted in bold. Th, T helper; Tc, T cytotoxic; TCR, T cell receptor; NK, natural killer; NKT, natural killer T, Treg, T regulatory. Data are expressed as mean ± SEM (*n* = 12 animals/group). Statistical differences: * *p* < 0.05 compared to the CON; # *p* < 0.05 compared to the RV group (by post hoc Dunn’s multiple comparison test).

Lymphocyte Populations in Spleen (%) ^1^	CON	RV	Rv + EV-EcN	Rv + EV-EcoR12
**1. B cells (CD45+)**	29.30 ± 1.21	25.17 ± 2.13	30.73 ± 1.59	27.81 ± 2.08
**2. TCRαβ+ cells**	12.48 ± 1.03	12.20 ± 1.57	15.00 ± 2.01	16.93 ± 1.75
2.1 Th cells (TCRαβ+ CD4+ NK-)	60.45 ± 1.42	55.46 ± 1.31 *	55.16 ± 1.78 *	54.89 ± 2.56 *
2.2 Tc cells (TCRαβ+ CD8+ NK-)	25.65 ± 0.84	30.23 ± 1.1 *	29.97 ± 0.8 *	31.72 ± 2.01 *
2.3 Tc/Th ratio	0.44 ± 0.02	0.54 ± 0.05 *	0.55 ± 0.04 *	0.56 ± 0.03 *
**3. TCRγδ+ cells**	1.56 ± 0.05	1.5 ± 0.17	1.986 ± 0.11 * #	2.00 ± 0.21 * #
3.1 TCRγδ+ CD8+	76.54 ± 1.01	75.99 ± 2.43	81.60 ± 1.77 *	85.43 ± 1.48 * #
3.2 TCRγδ+ CD8-	8.26 ± 0.26	9.68 ± 1.12	7.77 ± 0.59	6.78 ± 0.74
**4. NKT cells**	1.88 ± 0.1	1.95 ± 0.28	2.45 ± 0.36	2.67 ± 0.19 * #
4.1 NKT CD8+	87.82 ± 0.75	89.41 ± 0.8	89.25 ± 1.24	89 ± 1.15
4.2 NKT CD8-	5.21 ± 0.3	4.16 ± 0.52	5.05 ± 0.68	4.83 ± 0.67
**5. NK cells**	5.13 ± 0.16	4.21 ± 0.24	5.03 ± 0.34	4.11 ± 0.38
5.1 NK CD8+	22.64 ± 1.18	28.21 ± 4.48	34.54 ± 3.15 * (# *p* = 0.09)	43.78 ± 3.14 * #
5.2 NK CD8-	36.44 ± 0.82	38.53 ± 2.74	34.53 ± 1.91	31.25 ± 2.67
**6. Treg cells**	45.17 ± 3.48	42.33 ± 5.11	45.81 ± 3.46	45.28 ± 3.40

^1^ Results are presented as the percentage of positive cells in the lymphocyte population selected according to their forward-scatter characteristics (FCs) and side-scatter characteristics (SSCs) (Figure S3) or in a particular main lymphocyte population.

## Data Availability

The original contributions presented in the study are included in the article/Supplementary Materials. Further inquiries can be directed to the corresponding author.

## Supplementary Materials

The supporting information can be downloaded at: https://www.mdpi.com/article/10.3390/ijms25021184/s1.
