# Supplementary material for: Microbiota-Derived Extracellular Vesicles as a Postbiotic Strategy to Alleviate Diarrhea and Enhance Immunity in Rotavirus-Infected Neonatal Rats
Source: Int J Mol Sci. 2024 Jan 18;25(2):1184. doi: 10.3390/ijms25021184 (PMC10816611; doi:10.3390/ijms25021184)
Supplement: Supplementary file 1 [file ijms-25-01184-s001.zip › ijms-2822677-supplementary.pdf]

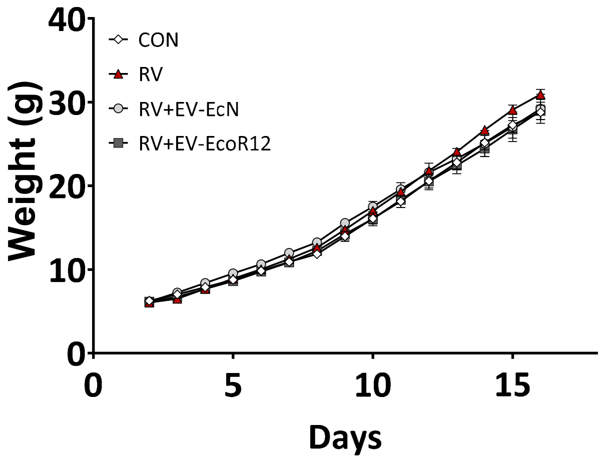

Figure S1. Control of animal weight (g) along the infection/ intervention period. Body weight was daily recorded for the four indicated experimental groups. Results are expressed as mean ± SEM (n = 12-24 animals/group). No statistically significant differences were observed between groups (by post hoc Dunn's multiple comparison test). The experimental groups were: CON: control group, RV: rotavirus group, RV+EV-EcN: rotavirus group receiving EcN EVs, RV+EV-EcoR12: rotavirus group receiving EcoR12 EVs.

Table S1. Growth associated variables.

	**Variable**	**CON**	**RV**	**RV+EV-EcN**	**RV+EV-EcoR12**
Day 8	Body length (naso-anal, cm)	6.87 ± 0.11	7.00 ± 0.07	6.98 ± 0.08	6.91 ± 0.12
Day 8	Body mass index (g/cm^2)	0.26 ± 0.01	0.26 ± 0.02	0.25 ± 0.01	0.26 ± 0.02
Day 16	Body length (naso-anal, cm)	9.32 ± 0.13	9.42 ± 0.09	9.32 ± 0.24	9.47 ± 0.23
Day 16	Body mass index (g/cm^2)	0.33 ± 0.01	0.33 ± 0.02	0.33 ± 0.02	0.32 ± 0.02

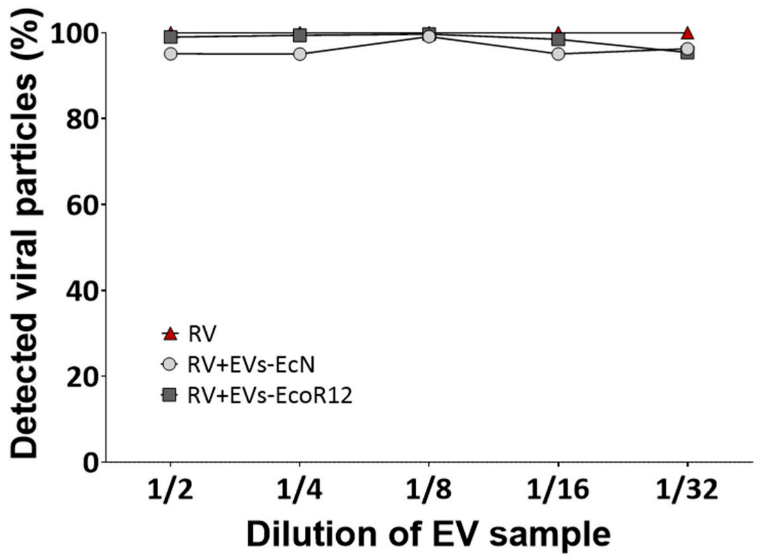

Figure S2. In vitro blocking activity of EcN and EcoR12 EVs on the immunological detection of SA-11 RV particles. RV was diluted in PBS-Tween at a final concentration of 5.8 × 10^7/mL. Stock samples of EcN EVs and EcoR12 EVs (40 μg/mL) were serially diluted and preincubated with the RV (1:1) for 30 min. An aliquot of non-preincubated RV solution was assayed a control. Free, unblocked viral particles were quantified by ELISA. Results are expressed as the percentage of detected viral particles compared to the control.

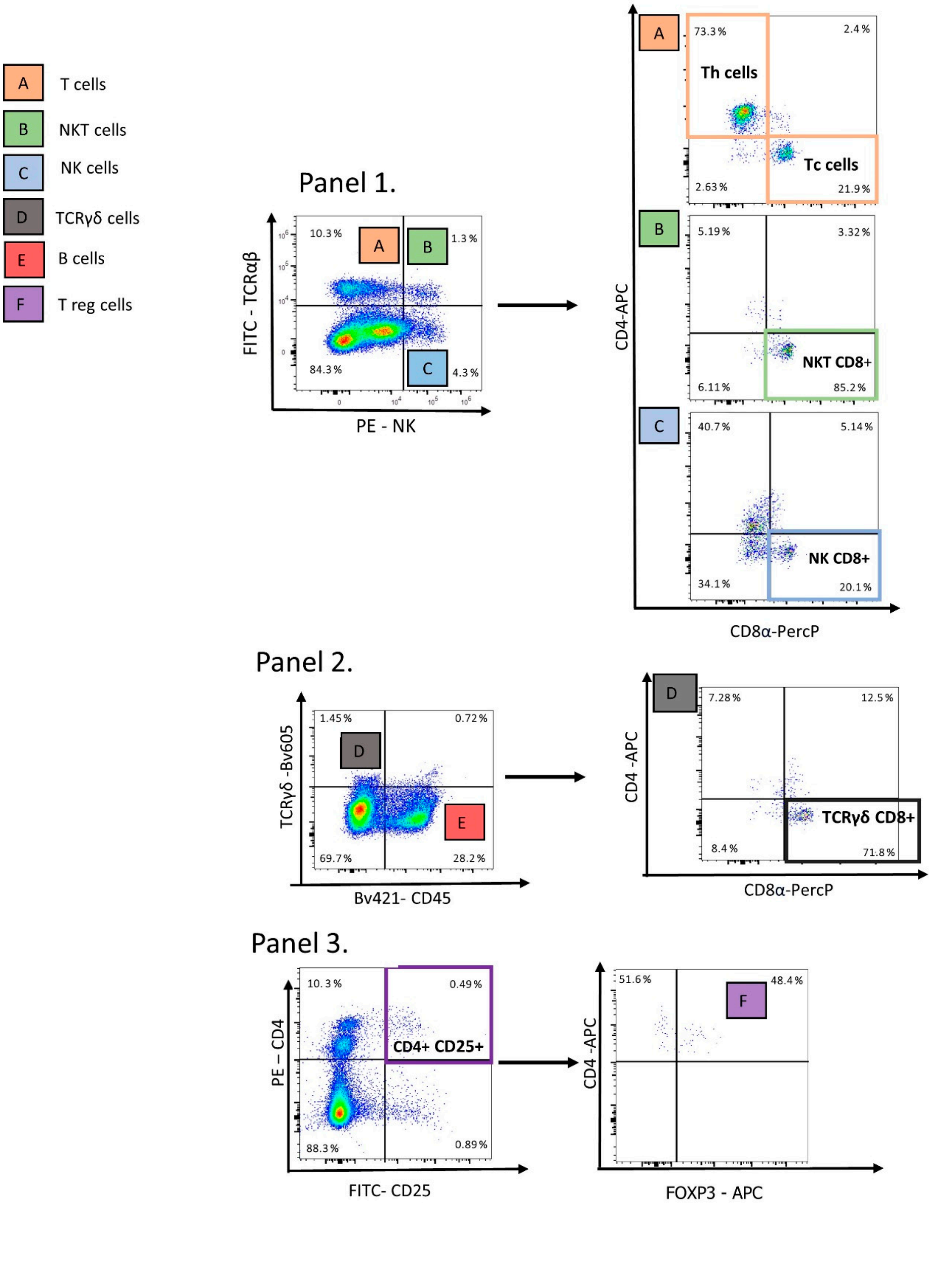

Figure S3. Flow cytometry panel design for the analysis of lymphocytes isolated from spleen samples at the last day of the experimental period (day 16). Representative dot plots of the the gates designed for the analysis the different lymphocyte subtypes are shown. The analyzed lymphocyte populations were: **Panel 1**: Th cells (TCRαβ+ CD4+ NK-), Tc cells (TCRαβ+ CD8+ NK-), NKT cells (TCRαβ+ NK+ CD8+), and NK cells (TCRαβ- NK+ CD8+. **Panel 2:** B cells (CD45RA+) and the T subset population expressing the receptor TCR-1 (TCRγδ+, classified in CD8+ and CD8- subsets). **Panel 3:** Treg cells (CD4+ CD25+ FoxP3+). Abbreviations: Th, T-helper; Tc, T-cytotoxic; NK, Natural Killer; NKT, Natural Killer T; Treg, regulatory T cells.
